# Supplementary material for: Use of hyperbaric oxygen therapy for preventing delayed neurological sequelae in patients with carbon monoxide poisoning: A multicenter, prospective, observational study in Japan
Source: PLoS One. 2021 Jun 18;16(6):e0253602. doi: 10.1371/journal.pone.0253602 (PMC8213185; doi:10.1371/journal.pone.0253602)
Supplement: Attachment — Submitted filename: Comments to Reviewers.docx [file pone.0253602.s002.docx]

We are grateful to the reviewers for their critical comments and useful suggestions, which have helped us to improve our paper considerably. As indicated in the responses that follow, we have taken all these comments and suggestions into account in the revised version of our paper. In the manuscript, the revised text is shown in red font. We have also provided the page and line numbers corresponding to the revisions in the text.

**Reviewer 1**

**1．The authors have made an effort to elaborate the patients’ initial conditions. However, there are important differences in the two groups that may have affected outcomes. For example, there were differences in the percentages of patients who lost consciousness (42.3% vs. 48.0%, NBO_2_ vs. HBO_2_), SpCO at the scene (26.4% vs. 30.1%). These differences were not statistically significant but that does not exclude a possible effect on outcome. In this case the authors used multivariable logistic regression to sort that our, however they need to state how they chose the variables that were in their regression model. It would be helpful to have a table that includes both univariable and multivariable risk factors for outcome.**

**Response:**

We have added the results of the univariate regression analyses of all the factors to Tables 5 and 6. The factors associated with DNS and unimproved PCD in previous reports [12–17] were included as variables in the multivariable logistic regression models. Because of the small numbers of patients whose SpCO at the scene (*n* = 8 in the NBO_2_ group and *n* = 40 in the HBO_2_ group; Table 1), SpCO at the scene was not included in the univariate or multivariable logistic regression analyses.

**2. Another important factor that suggests selection bias is that only 44% of patients in the NBO_2_ group were transferred to hospitals where HBO_2_ was available. It is therefore plausible that initial evaluation at those hospitals triaged to NBO_2_ those patients who were a priori expected to do well without HBO_2_, thus enhancing the outcome of the NBO_2_ group. It is also conceivably there may have been a selection bias on the part of the EMS to send less severely affected patients to NBO_2_ hospitals. Please could the authors comment. Among those patients who were referred to a hospital with hyperbaric facilities, it would be useful for information on the indications for treatment in that subgroup.**

**Response:**

There may be a selection bias because only 44% of patients in the NBO_2_ group were transferred to hospitals where HBO_2_ therapy was available. Among these, mildly affected patients may have received NBO_2_ therapy, although there was no significant difference in the COHb levels of the NBO_2_ group between patents transferred to institutions that offered only NBO_2_ therapy and patients transferred to institutions that also provided HBO_2_ therapy (20.9 ± 10.0% and 17.2 ± 10.1%, respectively, *P* = 0.101). There may be a selection bias arising from the EMS, as you mentioned, although some emergency centers did not have HBO_2_ chambers. We have added an explanation of this to the limitations (page 21, line 414) and the COHb levels to the results (page 9, line 187).

**3. The fact that SpCO wat the scene was 26.4% (NBO_2_) and 30.1% (HBO_2_), yet despite long times between exposure and hospital arrival (average of around 4 half times if the patients were breathing oxygen), COHb was decreased only to 19.3% and 18.7% in the two groups. Please could the authors comment**.

**Response:**

In our study, the numbers of patients with SpCO data were low (*n* = 8 in the NBO_2_ group and *n* = 40 in the HBO_2_ group; Table 1). This may help explain why COHb did not decrease enough on admission.

**4. In addition for the potential for selection bias, it is also possible that the relatively low numbers of adverse outcomes indicates that the study is underpowered to detect a difference. DNS was assessed either during a hospital visit or by phone. The relatively low rate of sequelae in this study compared with previous reports (e.g. Gorman et al, Anaesth Intensive Care. 1992;20:311-6, refs. 1 and 4) may have been because phone assessment is less sensitive than formal neuropsychological testing.**

**Response:**

We had previously described this limitation in the discussion section. We have added more text regarding this point in the discussion (page 16, line 300).

**5. There were differences between groups regarding CT findings, which should be described, along with information on abnormal MRI findings. Previous studies have observed imaging abnormalities as risk factors for DNS (e.g. Jeon, et al. JAMA Neurol. 2018;75:436-443). It would be helpful to include an analysis of the subset of patients with CT or MRI abnormalities should be included.**

**Response:**

We have added more text to the discussion describing the abnormal CT or MRI findings (page 18, line 360). However, because the amount of data was limited, we could not include the incidence of abnormal CT or MRI findings as a variable in the multivariable logistic regression models to identify factors associated with the incidence of DNS and unimproved PCD.

**6. In any retrospective analysis it is impossible to account for therapeutic decisions made based upon real-time assessment of patients, for example the observation that a greater number of HBO_2_ sessions in the first 24 hours was associated with a greater incidence of DNS. From this, the authors conclude: “Therefore, multiple rounds of HBO_2_ therapy should be administered with caution, because it is possible that symptoms will worsen.” Of the two factors here (number of treatments and clinical outcome), it is important to consider which one is cause and which one is effect. Contrary to the authors’ conclusion, it is more plausible that additional HBO_2_ was administered to patients who were not responding well.**

**Response:**

We have added text regarding this point to the discussion (page 18, line 355).

**7. The following is an overstatement: “Moreover, Scheinkestel et al. [6] reported harmful effects of HBO2 therapy in patients suffering acute CO poisoning.” It is also noteworthy that while this study reported fewer sequelae with normobaric vs. hyperbaric oxygen, if their protocol is to be the basis for treating CO poisoning, then 3-6 days of inpatient treatment are required.**

**Response:**

We have deleted this sentence.

**8. The following statement, “Experimental data have shown that HBO2 induces oxidative stress in healthy rat brains, measured as the lipid peroxidation products in brain cortex homogenates [24–26]” should be amended to include the observation that in CO poisoning HBO2 actually reduces oxidative stress (Thom SR. Toxicol Appl Pharmacol 1993;123:248-56). It is also of note that other animal studies of CO poisoning have revealed other beneficial effects of HBO2, including inhibition of leukocyte beta-2 integrins (reference 28), reversal of CO-cytochrome c oxidase binding (Adv Exp Med Biol 1989;248:747-54) and recovery of energy metabolism (J Clin Invest 1992;89: 666-72).**

**Response:**

We have added an explanation to the discussion and have reordered the discussion (page 17, line 225).

**9. The review of the literature in Conclusions suggests that all studies of HBO2 are equivalent, which they are not. Raphael’s and Annane’s studies were performed at 2 ATA, which as the authors point out is considered by many experts to be inadequate. In Raphael’s study the only comparison between NBO2 and HBO2 was in mild cases. Scheinkestel’s study utilized 3-6 days of therapy, which is usually considered impractical. On the other hand, studies at 2.5-2.8 ATA by Thom, Mathieu and Weaver have shown better outcome for HBO2. These details need to be included explicitly in the Discussion. The following statement pertains to a meta-analysis, “A recent meta-analysis of the therapeutic effects of different numbers of HBO2 sessions found that HBO2 therapy was associated with a lower risk of memory impairment than NBO2 therapy, but that two sessions of HBO2 therapy were associated with a higher risk of memory impairment than one session [30].” It is noteworthy that this meta-analysis also assumed equivalence of the various doses.**

**Response:**

The difference in the therapeutic pressure of the HBO_2_ therapy has been added to the discussion (page 18, lines 341–343, 347, and 351).

We are grateful to the reviewers for their critical comments and useful suggestions, which have helped us to improve our paper considerably. As indicated in the responses that follow, we have taken all these comments and suggestions into account in the revised version of our paper. In the manuscript, the revised text is shown in red. We have also provided the page and line numbers corresponding to the revisions in the text.

**Reviewer 2**

**Prior review: Major Issues:**

**1. How did providers determine who was to receive HBO_2_ therapy? This is not specified. I think if it was up to the provider, more analysis of the decisions/trends of the patients need to be identified. Did they use a general criteria for the trial? Did they go with a professional organization recommendation? The American College of Emergency Physicians does not provide specific recommendations, of note.**

**-> There was quite a bit of discussion around how many patients received what therapy. It was quite non standard but it was sufficiently reported here. Some were very strange (15 HBO_2_ sessions over a week), so not sure how to interpret these.**

**Response:**

The HBO_2_ therapy protocols were not consistent and depended on the practices at each participating institution, as we reported in reference 8.

**2. Mechanically ventilated patients were not included in the analysis. This is a major limitation. By my calculation, only 4.6% of the HBO_2_ patients required mechanical ventilation, whereas, 19.5% of the NBO_2_ group required mechanical ventilation. These patients were then not analyzed. This is a major limitation as these would be the most severely ill patients. Ideally, there is some sensitivity analysis that would include these patients. This may be why the delayed neurological sequelae are so low in the NBO_2_ group - the sickest were eliminated (19.5%). I do agree to not include patients that had an arrest upon arrival for this study.**

**-> The authors included mechanical ventilation patients in the analysis. This was a great improvement in the study and makes it much more applicable.**

**Response:**

Data about mechanically ventilated patients were added to manuscript, as you suggested.

**3. The definition of DNS is very vague. Page 7 refers to patients were "checked by a physician if any doubt". What does this mean? This needs to be listed very specifically how DNS was determined as it is a primary outcome of the study. If some of the list or discussion of the DNS determination algorithm needs to be in supplemental materials, that is fine. The exact method needs to be reported.**

**-> Clearer algorithm certainly. Still some limits. Is the screening questions asked ever been standardized or validated previously? With the 4x mental status exams, did the patient need to have an abnormality on any one of these or multiple tests? If it is only one deficit on a single test this could be a biased definition. The opposite is true as well – if very strict on defining (for instance, patients had to have all 4 with a deficit, then could be biased the other way (not reporting the DNS enough). I would clarify. This is much improved already though.**

**Response:**

DNS was diagnosed by a physician based on all the findings at the time of diagnosis, including a cognitive function test, such as the mini-mental state examination, the Wechsler adult intelligence scale, Hasegawa’s dementia scale-revised, the trail-making test, or the story recall test. We have revised the text in the data collection and analysis section (page 5, line 119).

**4. No reports on mortality in this group. I think it would be helpful. The size of the study and exclusion of cardiac arrest upon arrival patients does not warrant this being an outcome measure; however, the numbers should be reported.**

**-> better commentary on mortality overall**

**Response:**

In the present study, among 311 patients, there were three cases of CPAOA and three in-hospital deaths, but there were no patient deaths recorded during the follow-up period. However, 41 patients were lost to follow-up. We have added this to the discussion (page 20, line 391).

**5. Exposure distribution was unique - the most common causes of CO poisoning are generally motor vehicle exhaust and fires. Here, >50% of patients were exposed to burning charcoal. Is this the most common form of poisoning in Japan? This should be discussed more. Related, a lot more of the NBO_2_ group was exposed to fire, generally felt as the most severe form of exposure. This would provide that the effect seen is even more surprising.**

**-> much clearer. I would use term “intentional” not “suicide”. For the statistical comparison, I would do statistics for each exposure type (fire, exhaust, coal) instead of exposure in general.**

**Response:**

In previous reports, the most common causes of CO poisoning were internal combustion engines (Weaver et al., NEJM 2002;347: 1057-1067.) or gas water heaters (Annane et al., Intensive Care Med. 2011;3: 486-492). However, it was also reported that charcoal is a major cause of CO poisoning in Asia (Lin et al. J Chin Med Assoc. 2018;81: 682-690; Ku et al. Gen Hosp Psychiatry. 2010;32: 310-4.). Charcoal is still used for heating in northern Japan. We have also replaced the term “suicide” to “intentional” (page 8, Table 1) and have added data for each exposure type (fire, exhaust, coal) to Table 1.

**6. Why were the DNS events so low. Most reported prevalence is between 20-40% long term. Why was there only one DNS event in the NBO_2_ group? Is this because mechanical ventilated patients are excluded from the analysis? Again, I think this data needs to be reported at least in a sensitivity analysis if available. Else, should be listed as a major limitation.**

**-> Now addressed mechanical ventilation issue. Still quite low though. Any reason why you suspect? Perhaps points above about definition of DNS would be helpful to learn.**

**Response:**

The definition of DNS may partly explain why its incidence was low in this study. We have added a sentence to the discussion to describe this point (page 16, line 300).

**7. Why was the PaO2 higher in the HBO_2_ group? When was this lab taken? Was it prior to HBO_2_ initiation?**

**-> This was discussed a bit. Not sure why it is higher but not sure clinical meaningfulness.**

**Response:**

This might have been related to the greater number of patients affected by fires in the NBO_2_ group (Table 1). Patients affected by fires were more likely to suffer from smoke inhalation, and subsequently require intubation and ventilation (Table 3) because of a low P/F ratio. We have added an explanation of this to the discussion (page 119, lines 377 and 381).

**8. The issue of heterogeneity of therapy needs to be addressed as a major limitation - no standard intervention, multiple different pressures/number of hyperbaric treatments for the patients.**

**-> this was well discussed.**

**Response:**

We have added these points to the limitations (page 21, line 414).

**Applicable Minor issues:

1. 35% of facilities did not have access to HBO2 - this is actually a high percentage of HBO2 capable facilities. The US only has 250-350 nationwide. Is there a lot more hyperbaric centers in Japan or is it more that centers with HBO2 were more likely to enroll in this study? Is there data for how many centers in Japan?**

**-> related, I would include how many patients required intrafacility transfer for HBO2.**

**Response:**

At the start of the study, there were 568 institutions in Japan that had an HBO_2_ chamber, of which 115 institutions had a board-certified fellow of the Japanese Society of Hyperbaric and Undersea Medicine. We have described this in the methods section (page 4, line 98).

Unfortunately, we have no data about interfacility transfer for HBO_2_ therapy.

**2. Page 10, what does "period of oxygen administration" mean? I am confused by this term. Does that mean time before presentation to hospital on oxygen?**

**-> I am still not sure about this term. It is clearer how it is used now, but I think general length of stay in hospital is more meaningful. Could probably drop it. We can presume most of the patients received oxygen I think.**

**Response:**

This term refers to the period of oxygen administration during hospital stay; oxygen is not always administered for the entire hospital stay.

**3. When discussing controversy around HBO_2_ being effective or not, I would include the discussion of some of the large retrospective studies that suggest mortality or ADL benefit to HBO_2_:**

**a. Rose JJ, Nouraie M, Gauthier MC, et al. Clinical Outcomes and Mortality Impact of Hyperbaric Oxygen Therapy in Patients With Carbon Monoxide Poisoning. Crit Care Med. 2018;46(7):e649-e655. doi:10.1097/CCM.0000000000003135**

**b. Nakajima M, Aso S, Matsui H, Fushimi K, Yasunaga H. Hyperbaric oxygen therapy and mortality from carbon monoxide poisoning: A nationwide observational study. Am J Emerg Med. 2020;38(2):225-230. doi:10.1016/j.ajem.2019.02.009**

**-> I would still include this.**

**Response:**

We have escribed the survival rate and ADL of patients with CO poisoning in the discussion (page 20, line 391).

**Additional comments:**

**1. CK-MB is used for cardiac involvement. What was your cut off? How many had “High” values? Do you have troponin data available?**

**Response:**

We have added the number of the patients whose CK-MB was above the normal range to Table 1.

**2. What were the ECG abnormalities seen? 20-22% incidence is quite high.**

**Response:**

We have added the details of ECG abnormalities to Table 1.

**3. What were the CT abnormalities seen? Head CT or Chest CT?**

**Response:**

We have added the details of CT abnormalities to Table 1.

**4. What were MRI abnormalities seen, quite a few in the HBO_2_ group.**

**Response:**

We have added the details of MRI abnormalities to Table 1.

**5. Not sure you need a paragraph describing vital signs, we can read table**

**Response:**

We have deleted the text about vital signs.

**6. Why was there bed rest prescribed and what is the meaning of it? I did not think we prescribe this in clinical care anymore very much.**

**Response:**

We thought that bed rest might be related to the prevention of DNS. However, the length of bed rest seems to be affected by the period of mechanical ventilation in the present study. Therefore, we have deleted these data from Table 3.

**7. Give % mortality of overall cohort (included and excluded) when you report the number I think.**

**Response:**

We have included the number of deceased patients in the discussion, as you suggested (page 20, line 391).
