# Supplementary material for: Use of hyperbaric oxygen therapy for preventing delayed neurological sequelae in patients with carbon monoxide poisoning: A multicenter, prospective, observational study in Japan
Source: PLoS One. 2021 Jun 18;16(6):e0253602. doi: 10.1371/journal.pone.0253602 (PMC8213185; doi:10.1371/journal.pone.0253602)
Supplement: Attachment — Submitted filename: Comments to Reviewers R2.docx [file pone.0253602.s003.docx]

We are grateful to the reviewer 1 for his critical comments and useful suggestions, which have helped us to improve our paper considerably. As indicated in the responses that follow, we have taken all these comments and suggestions into account in the revised version of our paper. In the manuscript, the revised text is shown in red font. We have also provided the page and line numbers corresponding to the revisions in the text.

**Reviewer 1**

**1. Lines 325-338: the authors are attempting to make the point that HBO2-induced oxidative stress could the cause of worse outcome in the HBO2 group. In favor of this hypothesis they cite evidence that HBO2 induces oxidative stress. Of course it does. However, so does carbon monoxide exposure, and published evidence indicates that HBO2 reduces it (reference 26). The conclusion in lines 335-338 therefore needs to be amended.**

**Response:**

We have changed the sentence as you mentioned (page 18, line344-346).

**2. Lines 350-354: “A recent meta-analysis of the therapeutic effects of different numbers of HBO2 sessions, including a different range of therapeutic pressures from 2.0 to 2.8 ATA, found that HBO2 therapy was associated with a lower risk of memory impairment than NBO2 therapy, but that two HBO2 sessions was associated with a higher risk of memory impairment than one session [33].” This particular conclusion of Wang’s meta-analysis is based upon two studies (Raphael and Annane) in which sub-therapeutic HBO2 doses were used, and both mild and moderate sequelae (including memory impairment) were defined only by a patient questionnaire (no formal testing of memory); only 4 patients out of 170 had severe sequelae defined as objective abnormalities. All studies using HBO2 at 2.0 ATA have shown no benefit, while all studies using HBO2 at 2.8-3.0 ATA have shown benefit. Please add a caveat to this discussion.**

**Response:**

We had already discussed the difference of therapeutic effects between 2.0 ATA and 2.5 to 3.0 ATA in the 3^rd^ paragraph of the discussion (page16-17, line 315-333). We have changed the sentence of the discussion because our interpretation of the recent meta-analysis is incorrect as you mentioned (page 18, line 359-365).

**3. There were 171 patients in the HBO2 group, however the number who received a first HBO2 treatment (Table 2) totaled only 165. Please explain.**

**Response:**

In the six patients, HBO_2_ therapy were not administered during the first 24 h in 2 patients of the HBO_2_ group and the details of HBO_2_ therapy were unknown in 4 patents. This was the reason why the number of the patients received a first HBO_2_ treatment was 165.

We have added the contents to the results sessions of the revised manuscript (page 10, line 211-213).

**4. From the previous review: It would be helpful to include an analysis of the subset of patients with CT or MRI abnormalities. What were the outcomes for those individuals with brain abnormalities on either CT or MRI?**

**Response:**

Among 35 patients with abnormal findings in CT or MRI, DNS was observed in 2 (22.2%) and 8 (30.8%) patients in the NBO2 group (n = 9) and the HBO2 group (n=26), respectively. There was no significant difference in the incidence of DNS between the groups (P = 0.625). Unimproved PCD was observed in 2 (22.2%) and 6 (23.1%) patients in the NBO2 group and the HBO2 group, respectively. There was no significant difference between the groups (P = 0.958).

We have added the contents to the results sessions of the revised manuscript (page 13, line 252-257).

**5. The following points need to be addressed in a ‘Shortcomings’ section after the Discussion.

(1). This is not a randomized study, therefore, as pointed out in the previous review, the possibility of selection bias is large, both at the EMS level and within hospitals where HBO2 was available. Indeed, there is evidence that those patients who were treated with hyperbaric oxygen (HBO2) were more severely affected than those who were not. In addition to differences pointed out in the previous review (percentages of patients who lost consciousness, SpCO at the scene), the percentage of patients with abnormal CT, basal ganglia lesions) and MRI (basal ganglia lesions) was higher in the HBO2 group. The lack of statistical significance does not exclude a clinical relationship, particularly since fewer than 60% of patients who received HBO2 in this series were treated at a pressure considered to be therapeutic (≥2.4 ATA).

(2). There is no evidence in the manuscript that the assessors were blinded as to the treatment group (NBO2 vs. HBO2).

(3). An apparent dose-response between number of HBO2 treatments and incidence of delayed neurological sequelae (DNS) is incorrectly interpreted as evidence of possible harm due to HBO2. There is no statistical way to control retrospectively for a clinical observation related to improvement/lack of improvement during therapy. For example, a study of seriously ill infected patients might reveal a relationship between duration of antibiotic therapy and mortality. However on this basis it would be incorrect to conclude that antibiotic therapy is harmful; rather, longer antibiotic therapy was most likely prescribed because of lower clinical response to therapy in more severe infections.

(4). The relatively low statistical power to exclude an effect given the low rate of DNS and the fact that that many patients were not formally tested (the numbers are not stated) and 16% were lost to follow-up.**

**Response:**

We have briefly added the ‘Shortcomings’ section after the Discussion because majority of the contents have already addressed as limitations (page 21-22, line 435-441).

Further, we have added the sentence about non-blinded evaluator for DNS in the method session (page 5, line12-124).
